# Supplementary material for: HSP27 is a partner of JAK2-STAT5 and a potential therapeutic target in myelofibrosis
Source: Nat Commun. 2018 Apr 12;9:1431. doi: 10.1038/s41467-018-03627-9 (PMC5897330; doi:10.1038/s41467-018-03627-9)
Supplement: Supplementary file 1 — Supplementary Information(PDF 41502 kb) [file 41467_2018_3627_MOESM1_ESM.pdf]

SEVIN et al., supplementary data

“HSP27 is a partner of JAK2-STAT5 and a potential therapeutic target in myelofibrosis ”

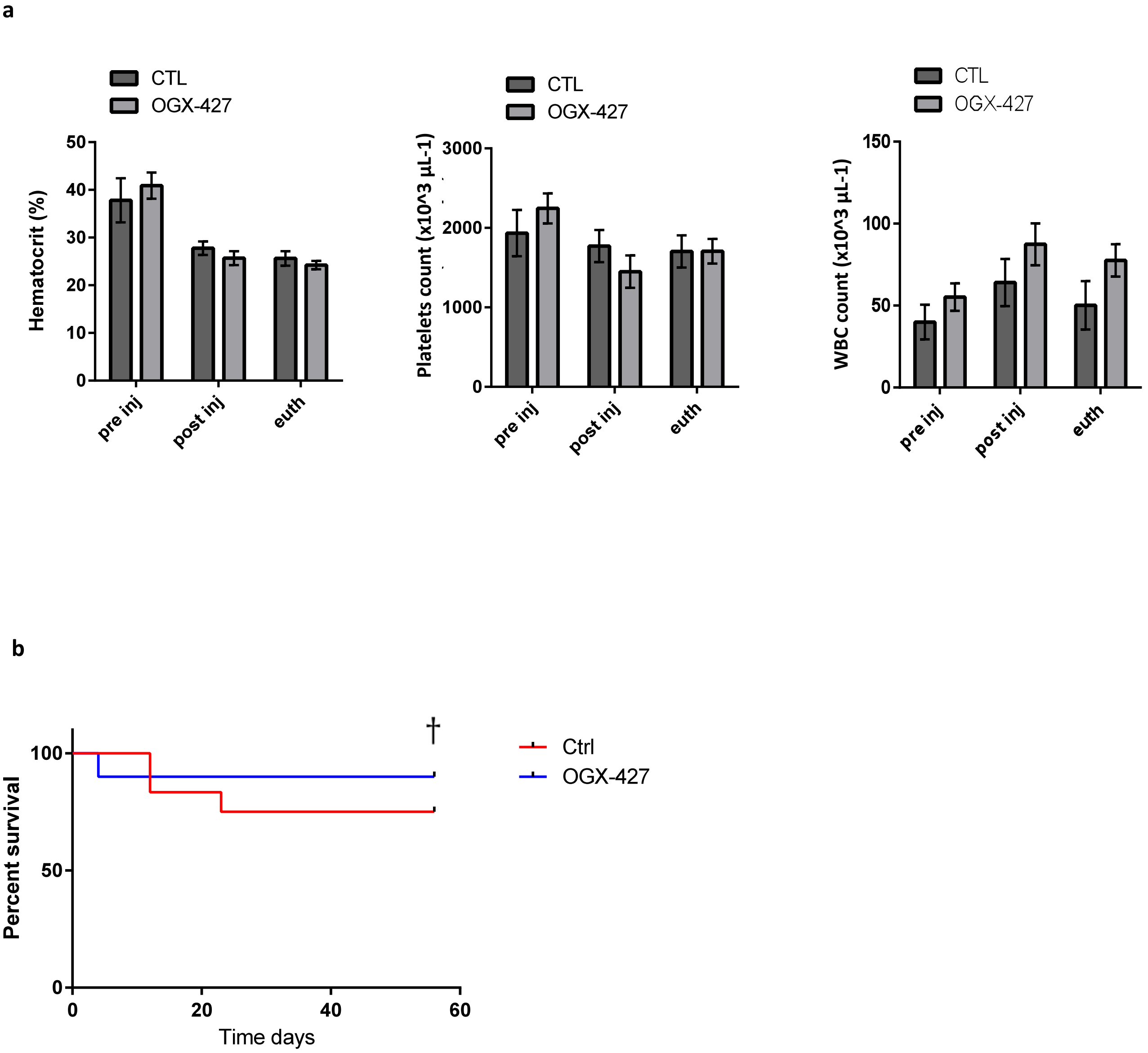

Supplementary Figure 1: (a) Blood cell parameters assessed in mice before and after OGX-427 (n=9) or CTL treatment (n=9) and on the day of euthanasia. WBC indicates white blood cells, error bars represent the standard errors of the mean. The kinetics of peripheral blood parameters were similar to those described in non-treated TPO^{high} mice[8], and OGX treatment had no significant effect. **(b)** A Kaplan-Meyer survival curve was done for the control (CTL, n=9) and treated group (OGX-427, n=9). Outcomes of the two treatments were compared using a log-Rank statistic analysis (p=0,32).

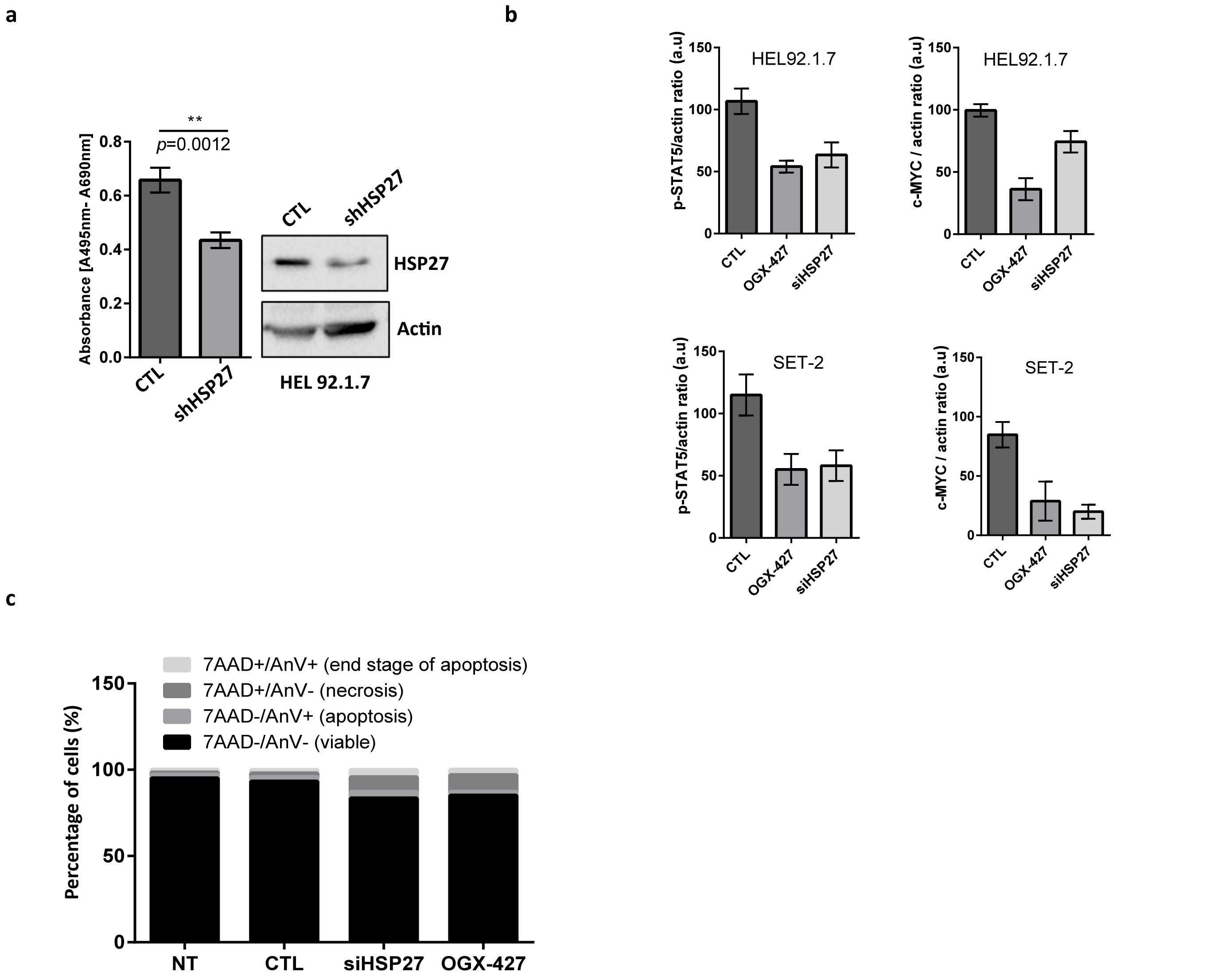

Supplementary Figure 2: (a) HEL92.1.7 cells were transduced with a lentiviral vector expressing either HSP27 shRNA or control vector (CTL) and GFP as a selection marker and 48h later cell proliferation was assessed using a XTT assay kit. (left panel) Bars represent cell proliferation percentages relative to shRNA CTL transfected cells from n=3 independent experiments. (Right panel) To assess the efficiency of shRNA HSP27, the expression level of HSP27 was determined by western-blot (12% SDS-PAGE). P-values were calculated using the Mann-Whitney test. **P < .01. Data are shown as means ±s.e.m. **(b)** Bar graphs show quantification of mean relative amount of the proteins analysed by western blot Fig. 3b (n=3 independent experiments), Data are shown as means ±s.e.m. **(c)** Representative histogram showing percentages of cell death from HEL92.1.7 cells transfected with OGX-427 or siRNA#1 HSP27 compared with CTL (NT = no transfected cells). Annexin V and 7AAD staining was analyzed 48h after transfection (n=2 independent experiments).

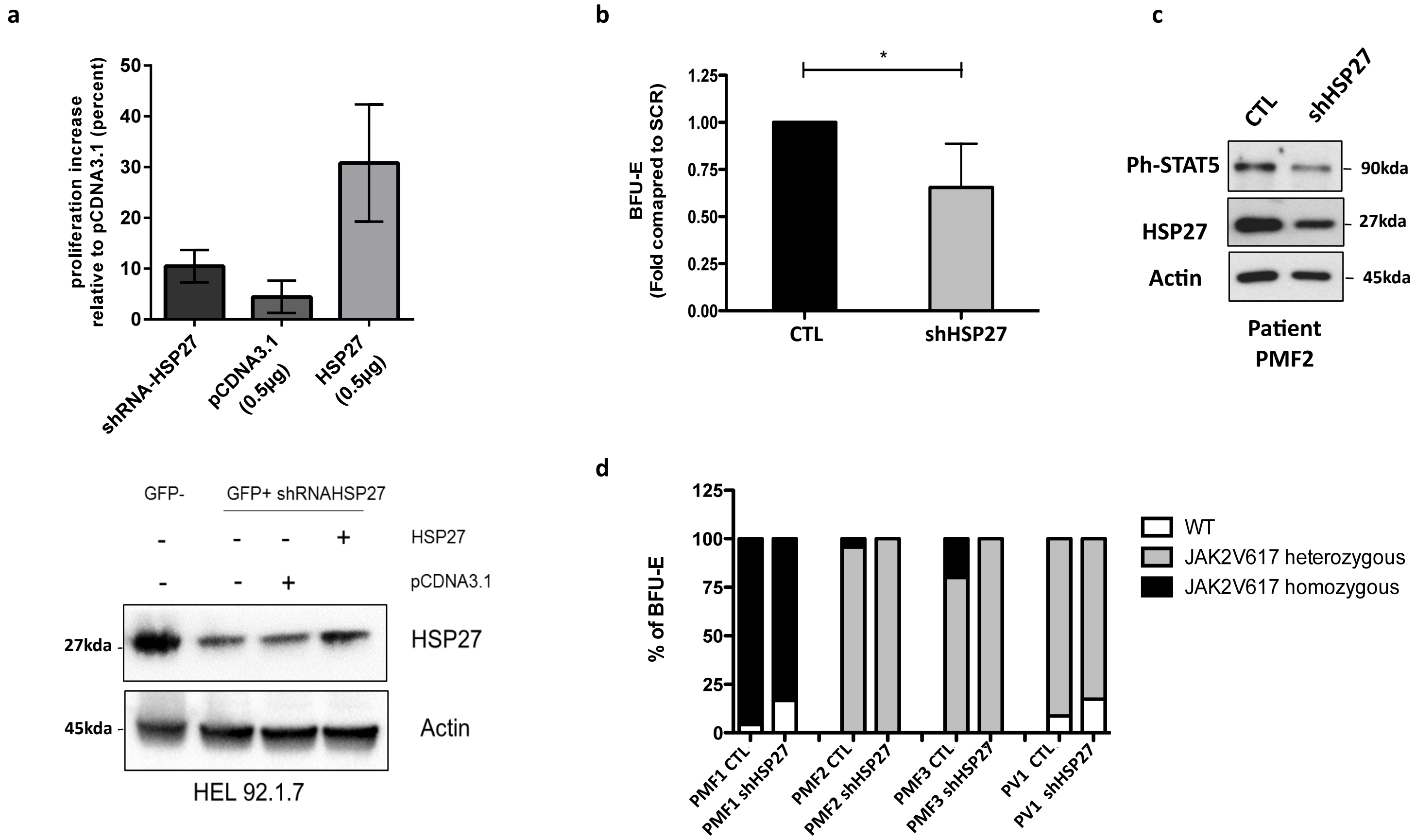

Supplementary Figure 3: (a) Rescue experiment in HEL92.1.7 cells. 0.5 millions cells were successively infected with a shRNA HSP27 for 72h, then transfected 24h hour later with a HSP27 plasmid. Upper panel, cell proliferation was assessed after 48h using a XTT assay kit. Bars represent cell proliferation percentages relative to pCDNA 3.1 transfected cells. Data are shown as means ±s.e.m; n = 2. Lower panel, to assess the efficiency of HSP27 transfection, the expression level of HSP27 was determined by western-blot (12% SDS-PAGE). **(b)** HSP27 knockdown decreases clonogenic potential of human erythroid progenitors (BFU-E) formation and specifically targets *JAK2V617F* clones in MPN patients CD34$^+$ from MPN patients (4 PMF and 1 PV) were transduced with lentiviral vector encoding shRNA-HSP27 (shHSP27) or control vector (CTL) and GFP. The CD34$^+$/GFP$^+$ were sorted 24 hours later and cells were plated in semisolid conditions (methylcellulose) BFU-E of the 4 PMF patients were counted 14 days later. Data are shown as means ±s.e.m.; n = 4; *P < 0.05, Student's *t* test. **(c)** Western blot analysis of HSP27 and ph-STAT5 in primary cells from patient PMF2. Actin served as the loading control. **(d)** Figure represents the fold decrease in shHSP27 knockdown condition compared to CTL vector. Approximately 18–24 colonies were plucked in each condition and analysed for *JAK2V617F* (1 PV, 3 PMF). The percentage of colonies (normal(WT)/heterozygous/ homozygous) is indicated in the figure for each patient; n = 4.

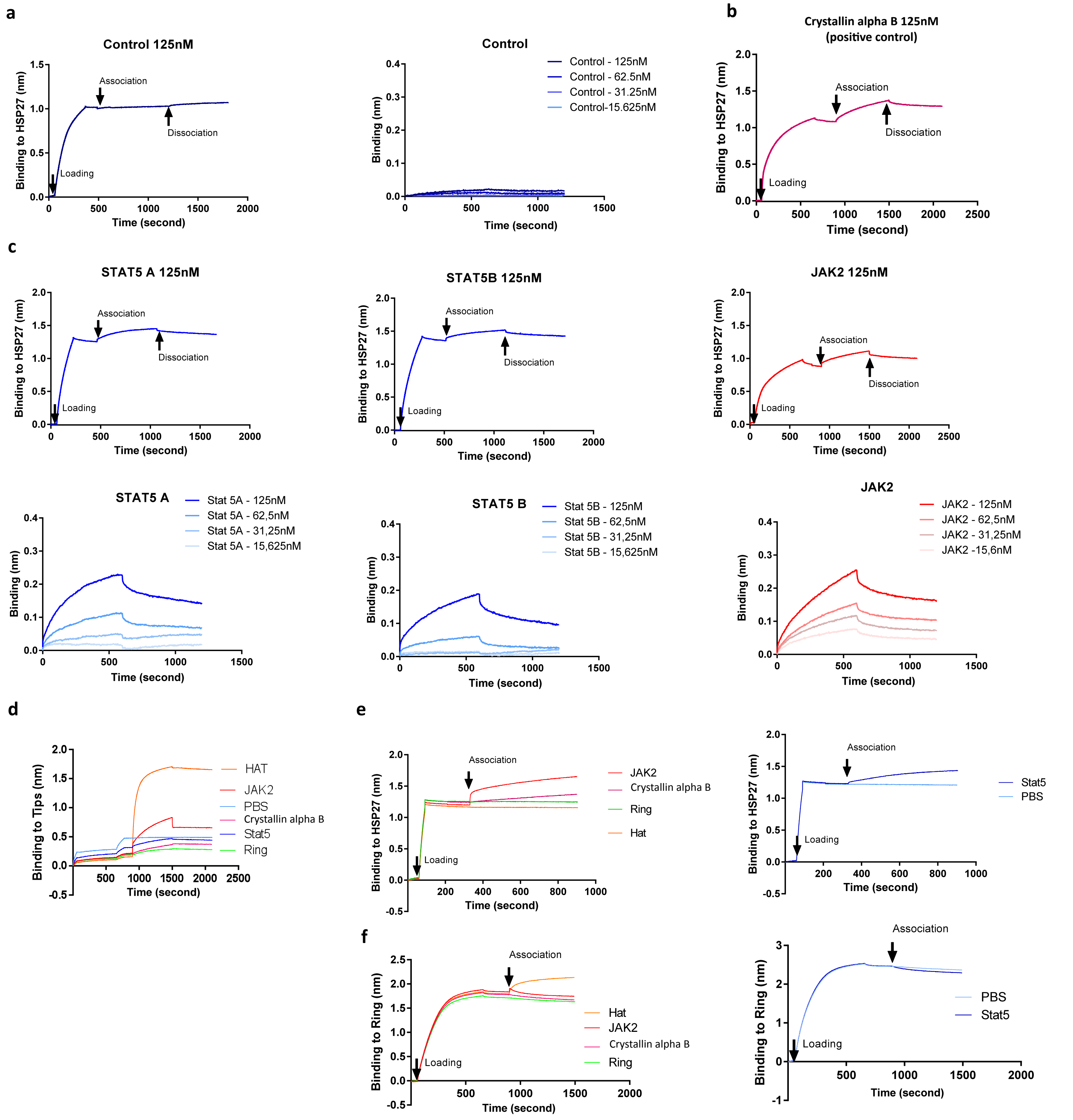

Supplementary Figure 4: (a) The binding of the recombinant RING domain of the CBP protein used as the negative control to immobilized biotinylated HSP27 was determined by biolayer interferometry (control) (n=3 independent experiments). **(b)** The binding of recombinant alpha B crystallin to immobilized biotinylated HSP27, used as a positive control [30], was determined by biolayer interferometry (n=3 independent experiments). **(c)** Raw data for the loading step. The association and dissociation steps were done at 26°C as follow: Baseline = PBS - 60s; Loading = HSP 27 – 600s (the loading was stopped at 1nm), two successive Baselines = - 120s; Association step: - 600s; Dissociation step : - 600s. (n=3 independent experiments)**. (d-f)** Two assay run. The binding of recombinant JAK2, STAT5A/B, Crystallin alphaB, HAT and RING domains of CBP at 125nM to immobilized biotinylated HSP27 or RING domain (irrelevant protein) was determined by biolayer interferometry **(d)** No specific binding on streptavidin biosensors **(e)** Biotinylated recombinant HSP27 was used as a ligand and immobilized at 10 µg ml^{-1} on streptavidin biosensors after dilution in PBS. **(f)** Biotinylated recombinant RING domain of CBP was used as a ligand and immobilized at 10 µg ml^{-1} on streptavidin biosensors after dilution in PBS. NB: HAT and RING domain are known to interact with each other.

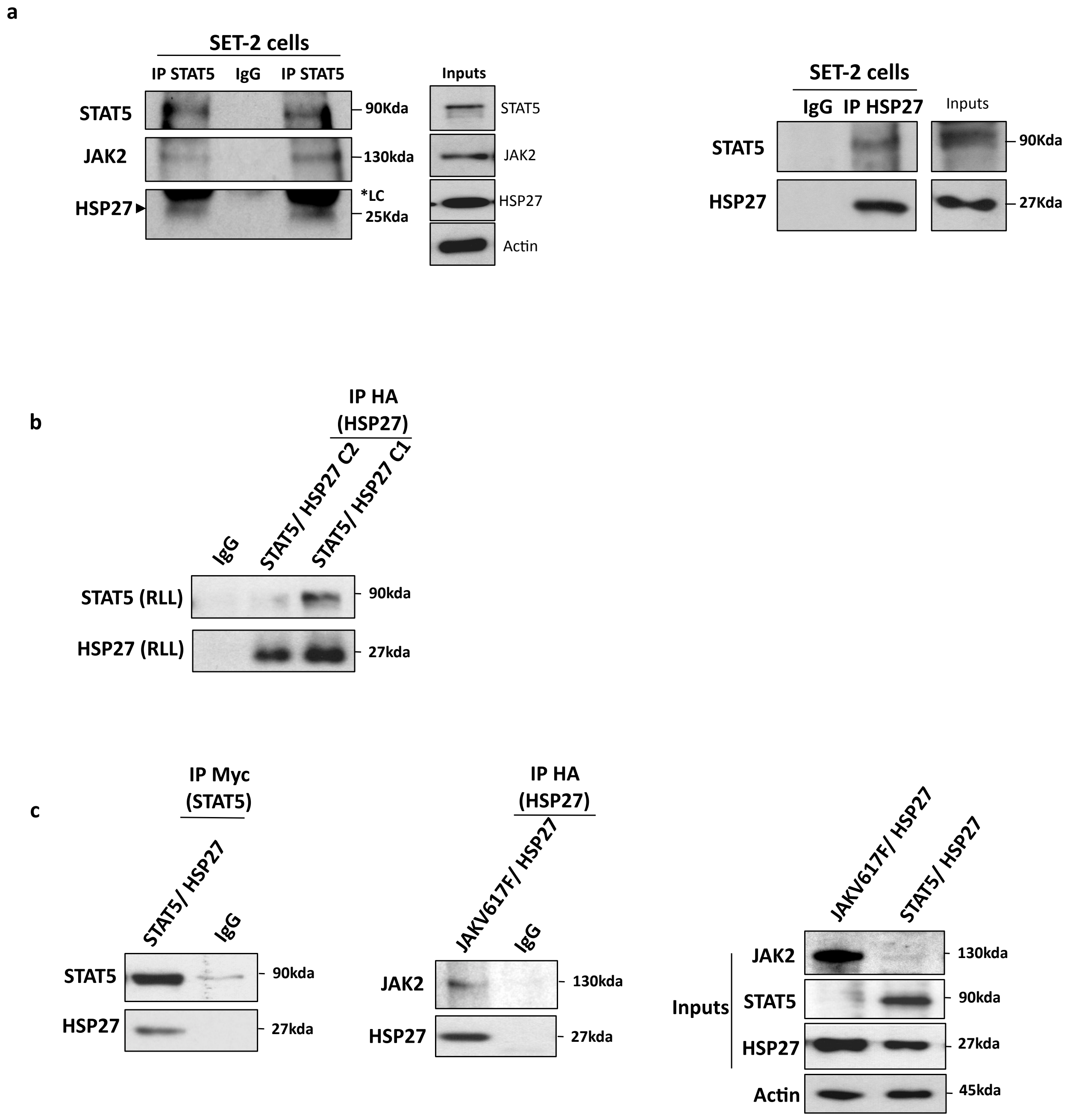

Supplementary Figure 5: (a) Immunoprecipitation from SET-2 cell extracts of endogenous STAT5 (left panel) or HSP27 (right panel) was followed by immunodetection of endogenous HSP27 and JAK2 (left panel) or STAT5 (right panel). Inputs: proteins in total cell lysates. IP IgG: immunoprecipitation with a non-relevant antibody (n=2 experiments). **(b)** Recombinant proteins Myc-tagged STAT5a and HA-tagged HSP27 produced in the TNT rabbit reticulocyte lysate (RLL) were incubated 15 min at 37°C and then subjected to immunoprecipitation using an HA antibody (HA-HSP27) followed by an immunodetection of Myc-tagged STAT5 (n=2 independent experiments). C1: 1 volume of HSP27; C2: 2 volumes of HSP27. **(c)** HEK293T cells were transfected with HA-tag HSP27 and Myc-tagged STAT5a or JAK2 and 24h later, an Immunoprecipitation on HEK293T cells extract was performed using anti-HA antibody (HA-HSP27) or anti-Myc antibody (Myc-STAT5a) followed by an immunoblot using a STAT5 or JAK2 antibody, respectively. Inputs: proteins in total cell lysates. IP IgG: immunoprecipitation with a non-relevant antibody (IgG mouse) (n=3 independent experiments).

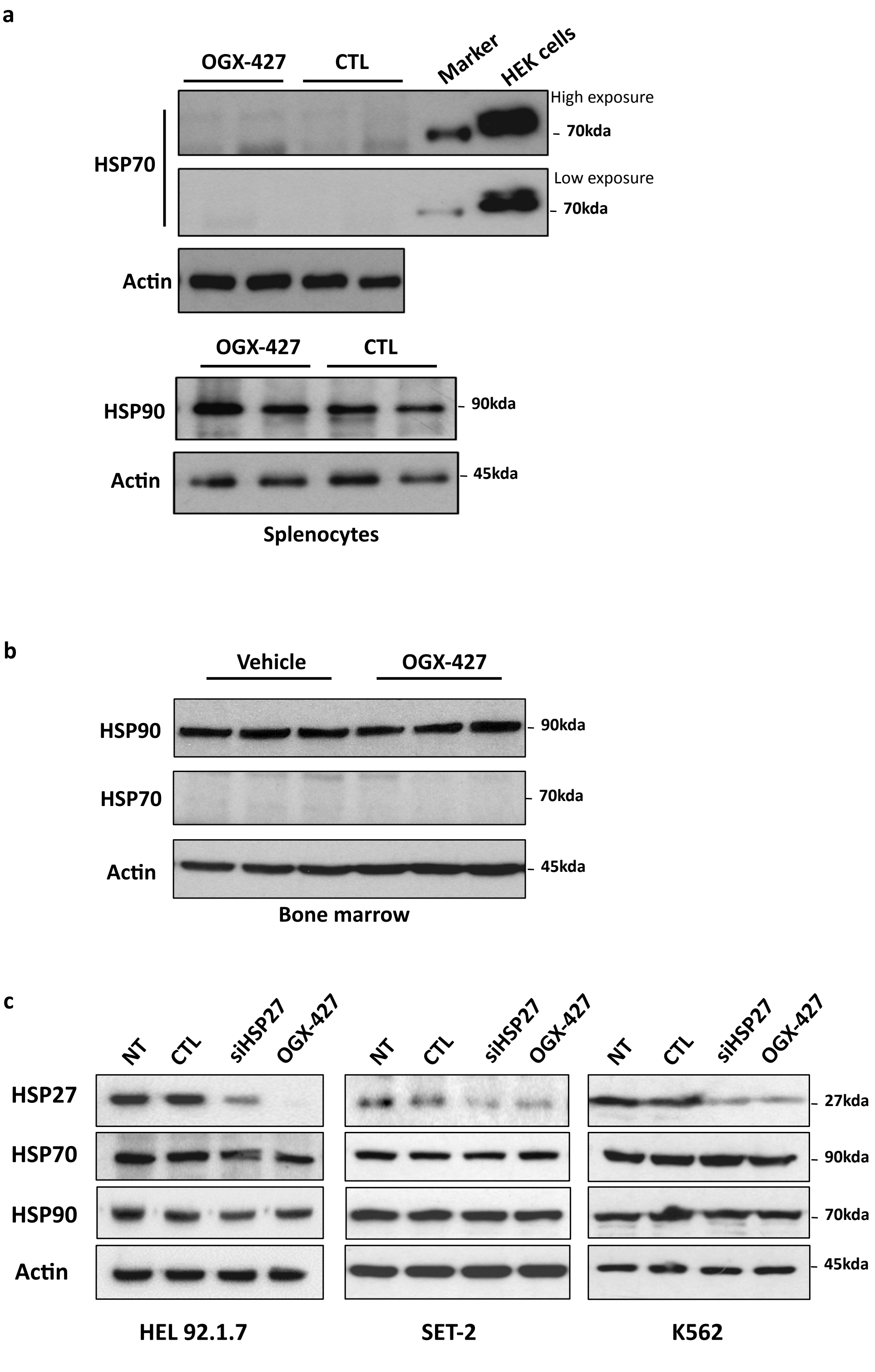

Supplementary Figure 6: (a) Western blot analysis of HSP70 and HSP90 in splenocytes (whole cell lysates) 24 days after treatment of TPO^{high} MF mice with OGX-427 or CTL (n=2 per group). Actin served as the loading control. HEK cells served as positive control. **(b)** Western blot analysis of HSP70 and HSP90 in bone marrow (whole cell lysates) 22 weeks after treatment of *JAKV617F* mice with OGX-427 or CTL (n=3 per group). Actin served as the loading control. **(c)** HEL92.1.7, SET-2 and K562 cells were transfected with HSP27 siRNA#1, OGX-427 or an oligonucleotide control (CTL) using the Amaxa Nucleofector silencing siRNA system (NT = no transfected cells). 48h later, the expression of HSP27, HSP70 and HSP90 were determined by western-blot (n=2). Actin was used as the loading control.

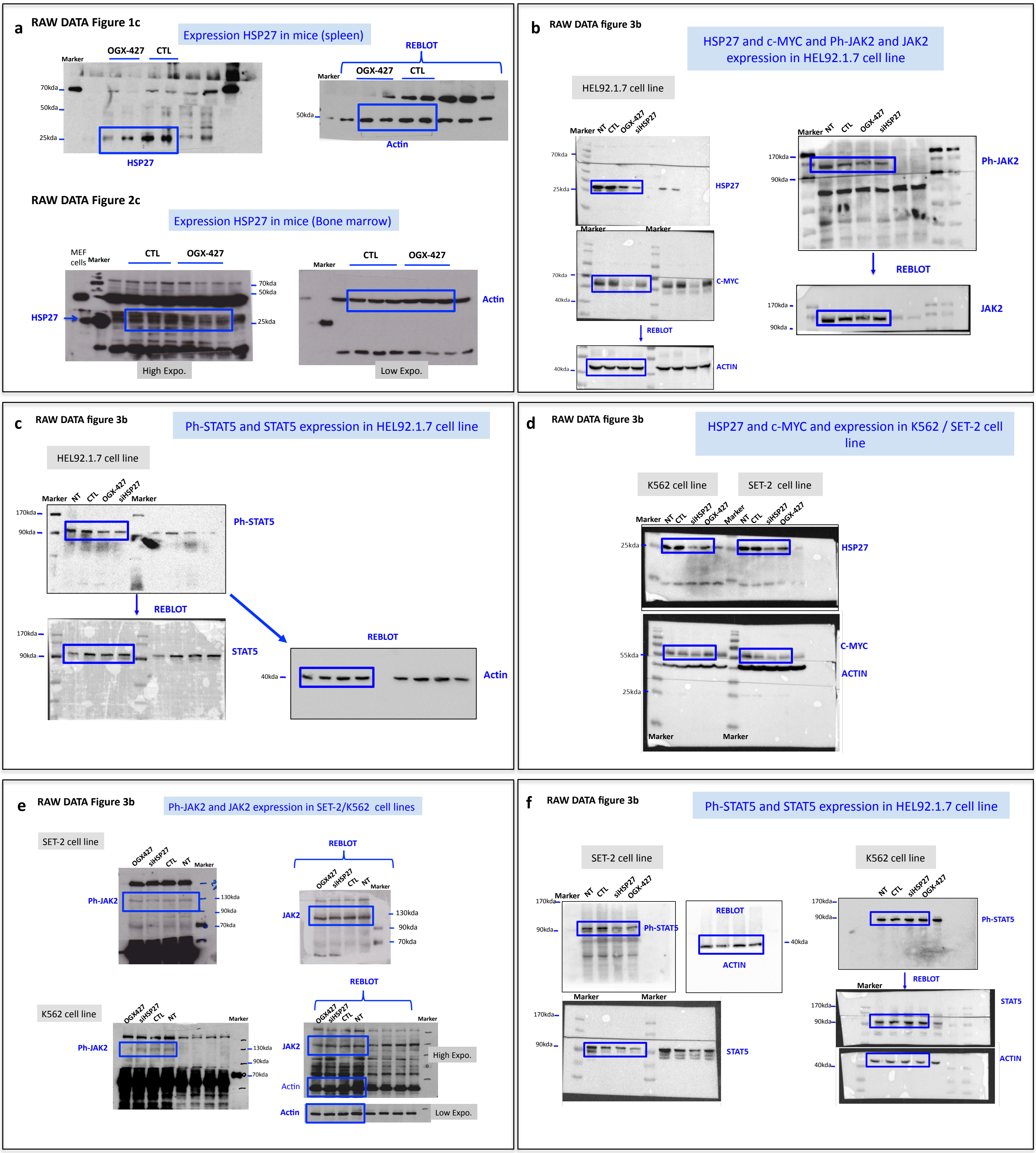

Supplementary Figure 7: Uncropped of Western blot analyses presented in indicated Figures (a) full blot from main Fig. 1 and 2 **(b-f)** full blots from main Fig. 3

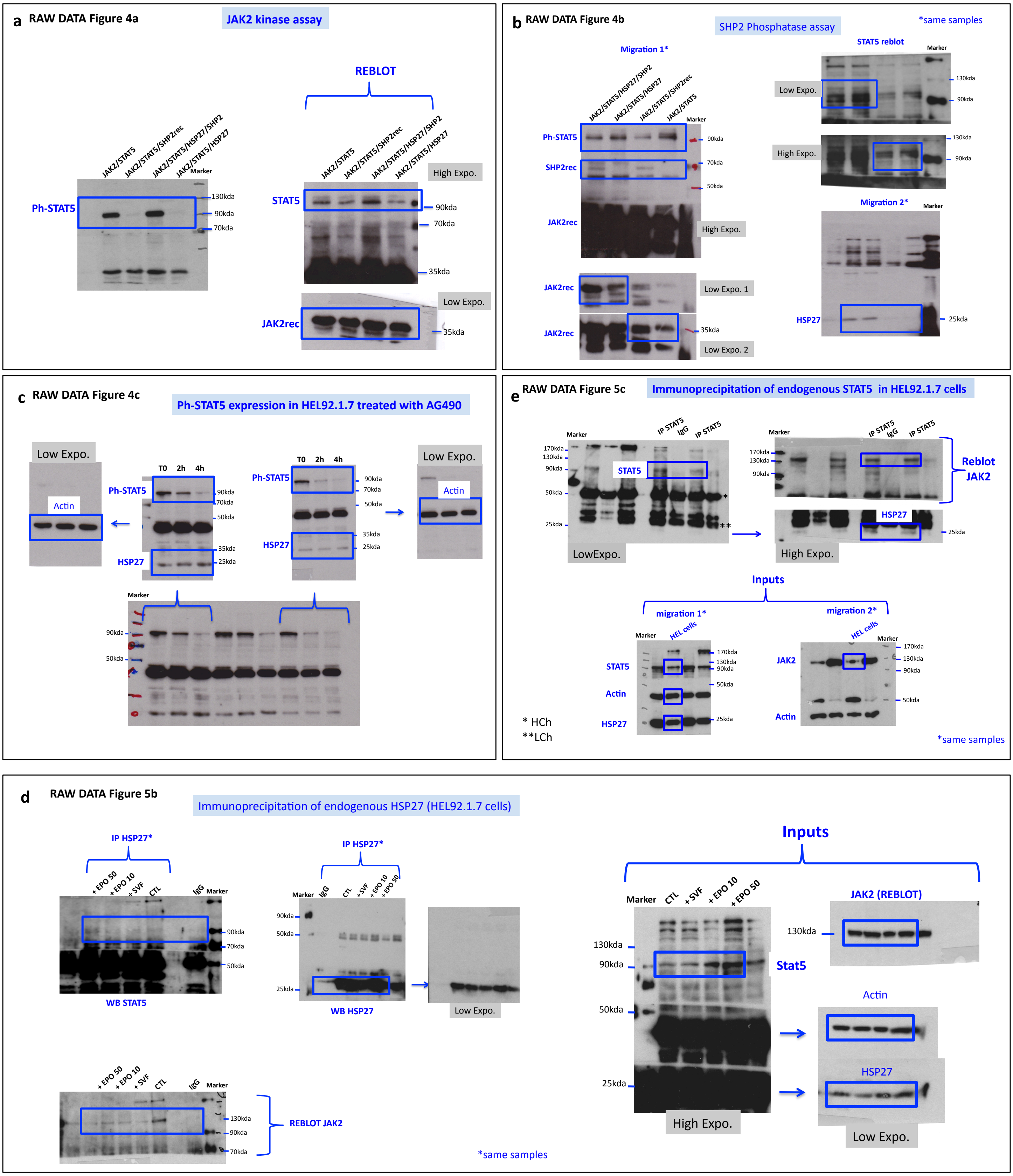

Supplementary Figure 8 : Uncropped of Western blot analyses presented in indicated Figures (a-c) full blot from main Fig. 4; **(d,e)** full blots from main Fig. 5

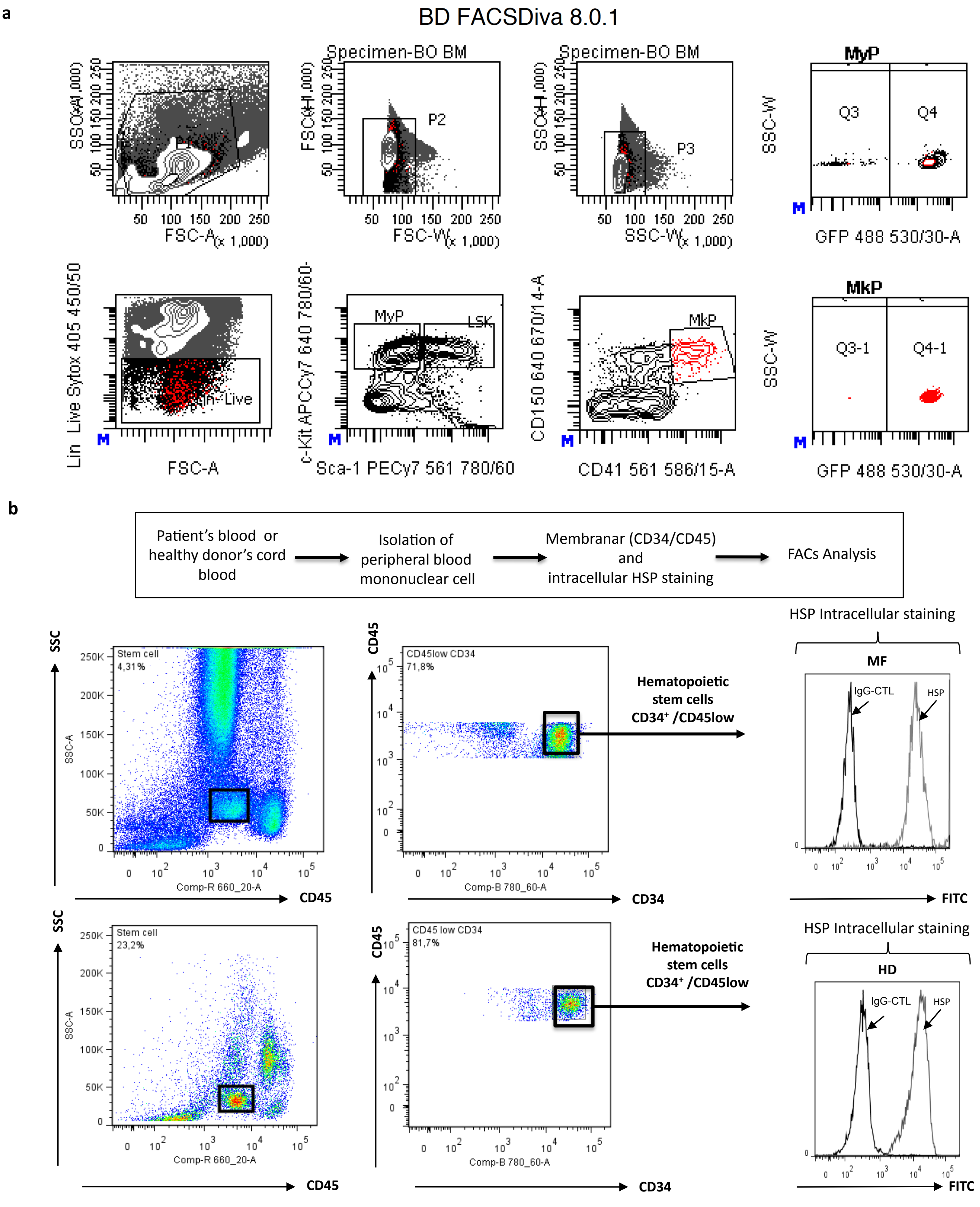

Supplementary Figure 9: (a) Quantification of megakaryocytes in bone marrow. Representative images of megakaryocyte cell-gating for the flow cytometry analysis are shown (n=5 mice per group). Bone marrow cells (FSC-A vs SSC-A; FSC-H vs FSC-W; SSC-H vs SSC-W, graphs 1-3, upper panel); Lin$^-$ live (Sytox negative) cells (first graph, lower panel); myeloid progenitor (MyP) population (c-kit$^+$, Sca-1$^-$, second graph, lower panel); megakaryocyte progenitor (MkP) population (CD150$^+$, CD141$^+$, third graph, lower panel); MyP or MkP cells gated based on GFP expression: GFP$^-$ (Q3 and Q3-1) and GFP$^+$ (Q4 and Q4-1) populations are shown (last graph, upper and lower panel, respectively). **(b)** Determination by FACs of intracellular HSP protein levels in Hematopoietic stem cells (CD34$^+$/CD45low) isolated from the blood of patients (MF, upper panel) or cord blood of Healthy donor (HD, lower panel). IgG CTL: Immunoglobuline control. Representative images of FACS gating are shown (number of sample analysed; MF n=11-18;HD: n=10-12).

Patients	Primary/secondary MF	MUTATION
#002	SMF	*JAK2*
#005	PMF	*JAK2*
#006	SMF	*JAK2*
#007	PMF	*JAK2*
#008	PMF	*CALR*
#009	SMF	*JAK2*
#010	PMF	*JAK2*
#011	SMF	*JAK2*
#012	SMF	*triple neg.**
#013	SMF	*CALR*
#014	PMF	*CALR*
#0017	SMF	*JAK2*
#0018	PMF	*CALR*
#0019	PMF	*JAK2 neg.***
#0021	PMF	*MPL*
#0022	SMF	*JAK2*
#0024	PMF	*JAK2*
#0026	SMF	*JAK2*
#0027	PMF	*JAK2*
#0028	PMF	*CALR*
#0030	PMF	*JAK2*
#0031	PMF	*JAK2*
#0032	SMF	*JAK2*

Supplementary Table 1: Description of the patient's mutation

List of the patients characterized by an increased level of HSP27 (>2ng ml^{-1}) compared to Healthy donor (HSP27 mean level =2,03 ng ml^{-1}) in sera and whose mutation was known. SMF: Secondary myelofibrosis, PMF: Primary myelofibrosis, *CALR*: Calreticulin, *MPL*: Thrombopoietin receptor. *no driver mutation, ***JAK2* mutation negative.
